# Effect of the MIND Diet Intervention on Cognition in Older Adults with Class 2 and 3 Obesity

**DOI:** 10.1093/geroni/igaf122.2160

**Published:** 2025-12-31

**Authors:** Shannon Halloway, Lisa Barnes, Neelum Aggarwal, Konstantinos Arfanakis, Frank Sacks, Klodian Dhana

**Affiliations:** University of Illinois Chicago, Chicago, Illinois, United States; Rush University Medical Center, Chicago, Illinois, United States; Rush University Medical Center, Chicago, Illinois, United States; Rush University Medical Center, Chicago, Illinois, United States; Harvard School of Public Health, Boston, Massachusetts, United States; Rush University Medical Center, Chicago, Illinois, United States

## Abstract

With increasing prevalence of obesity in the United States, dietary interventions that promote weight loss have high public health significance. In the recently completed clinical trial (i.e., MIND diet trial), both the MIND diet intervention and control diet groups received mild caloric restriction and showed clinically significant weight loss and improved cognition over 3 years. In the current study, we examined whether the effect of the MIND diet intervention on cognition differs across people with different body mass index (BMI) at enrollment. The MIND trial enrolled older adults without cognitive impairment but with a family history of dementia, BMI>25, and a suboptimal diet. A linear mixed-effect model was used to examine if BMI modified the effect of MIND on cognition, specifically annual change in a global cognition score derived from a battery of 12 neurocognitive tests. Participants (N = 604) were, on average, 70.4 years old, 65.5% female, and with a BMI of 33.9 kg/m2 at baseline. In a multivariable-adjusted model, there was a significant interaction between intervention x BMI x time (p= .011); as such, among individuals with BMI>35 kg/m2 (n = 213), those within the MIND diet group showed a slower rate of decline in global cognition by 0.040 (SE = 0.017, p=.018) standardized units per year when compared to people in the control diet group. In people with BMI< 35, there were no differences between the MIND diet and the control group in global cognition. Future work should examine underlying mechanisms that explain the cognitive benefits of the MIND diet among people with obesity.

